# Nutrient and Sensory Metabolites Profiling of *Averrhoa Carambola* L. (Starfruit) in the Context of Its Origin and Ripening Stage by GC/MS and Chemometric Analysis

**DOI:** 10.3390/molecules25102423

**Published:** 2020-05-22

**Authors:** Nehal S. Ramadan, Ludger A. Wessjohann, Andrei Mocan, Dan C Vodnar, Nabil H. El-Sayed, Sayed A. El-Toumy, Doha Abdou Mohamed, Zeinab Abdel Aziz, Anja Ehrlich, Mohamed A. Farag

**Affiliations:** 1 Chemistry of Tanning Materials and Leather Technology Department, National Research Centre, Dokki, Cairo 12622, Egypt; nehalsameh111@gmail.com (N.S.R.); nabil_17@yahoo.com (N.H.E.-S.); sayedeltomy@yahoo.com (S.A.E.-T.); 2 Leibniz Institute of Plant Biochemistry, Department Bioorganic Chemistry, Weinberg 3, D-06120 Halle (Saale), Germany; anja.ehrlich@ipb-halle.de; 3 Department of Pharmaceutical Botany, Faculty of Pharmacy, Iuliu Hatieganu University of Medicine and Pharmacy, 400372 Cluj-Napoca, Romania; mocan.andrei@umfcluj.ro; 4 Laboratory of Chromatography, Institute of Advanced Horticulture Research of Transylvania, University of Agricultural Sciences and Veterinary Medicine, 400372 Cluj-Napoca, Romania; 5 Department of Food Science, University of Agricultural Sciences and Veterinary Medicine, 400372 Cluj-Napoca, Romania; dan.vodnar@usamvcluj.ro; 6 Nutrition and Food Sciences Department, National Research Centre, Dokki, Cairo 12622, Egypt; dohamohamed@yahoo.com; 7 Pharmacognosy Department, College of Pharmacy, Cairo University, Kasr El Aini St., P.B. 11562 Cairo, Egypt; zeinab.kandil@pharma.cu.edu.eg; 8 Department of Chemistry, School of Sciences & Engineering, The American University in Cairo, New Cairo 11835, Egypt

**Keywords:** primary metabolites, *A*. *Carambola* L., Oxalidaceae, GC-MS, SPME, volatiles, diabetes type-2

## Abstract

*Averrhoa carambola* L. is a tropical tree with edible fruit that grows at different climatic conditions. Despite its nutritive value and reported health benefits, it is a controversial fruit owing to its rich oxalate content. The present study aimed at investigating aroma and nutrient primary metabolites distribution in *A. carambola* fruits grown in Indonesia, Malaysia (its endemic origin) versus Egypt, and at different ripening stages. Two techniques were employed to assess volatile and non-volatile metabolites including headspace solid-phase micro-extraction (HS-SPME) joined with gas chromatography coupled with mass-spectrometry (GC-MS) and GC-MS post silylation, respectively. Twenty-four volatiles were detected, with esters amounting for the major class of volatiles in Egyptian fruit at ca. 66%, with methyl caproate as the major component, distinguishing it from other origins. In contrast, aldehydes predominated tropically grown fruits with the ether myristicin found exclusively in these. Primary metabolites profiling led to the identification of 117 metabolites viz. sugars, polyols and organic acids. Fructose (38–48%) and glucose (21–25%) predominated sugar compositions in ripe fruits, whereas sorbitol was the major sugar alcohol (2.4–10.5%) in ripe fruits as well. Oxalic acid, an anti-nutrient with potential health risks, was the major organic acid detected in all the studied fruits (1.7–2.7%), except the Malaysian one (0.07%). It increases upon fruit ripening, including considerable amounts of volatile oxalate esters detected via SPME, and which must not be omitted in total oxalate determinations for safety assessments.

## 1. Introduction

The recently expanding attentiveness to functional foods, as well as the urgent need for assessment of their nutritive values and safety, warrants the development of advanced methods for their chemical analysis [1]. The complexity of plant matrices, chemical composition, and moreover the variation of food bioactive compounds based on geographical origin, genotype, agricultural practice, growing conditions, ripening stages and or processing methods are all reported [2] and known to affect functional food biological effects.

For centuries, *Averrhoa carambola* L. belonging to the family Oxalidaceae was recognized as being native to tropical Southeast Asia and cultivated throughout the tropics for its edible fruit [3], as well as for its decorative character. Nevertheless, its tree has been domesticated in other regions [4,5] such as Ecuador [6] and more recently in Egypt. Due to its vast distribution throughout different regions, *A. carambola* fruit acquired different common names that is ‘Yangtao’ in Chinese, ‘carambola’ or ‘starfruit’ in English [7], belimbing besi among locals in Malaysia [8]. 

The fruit exhibits two main types of taste, sweet and sour, with a complicated flavor combination that includes plum, pineapple, and lemon notes [3,9]. The mature fruit taste is characterized by being both sweet and juicy [10]. The fruit is widely used in Asian foods, and its juice is considered a popular thirst-quencher [8]. 

Considering its rich documented traditional uses, a tea is prepared from the fruit in Ayurvedic medicine for its pharmaceutical properties [11] to relieve indigestion, hemorrhoids, fever [10] headaches, vomiting, coughing, in addition to its use as an appetite stimulant, diuretic and antidiarrheal [12]. Pharmacological assays confirmed starfruit therapeutic effects viz. anti-inflammatory, antimicrobial, antifungal, antitumor, anti-ulcer, hypocholesterolemic, hypoglycemic and hypotensive effects [13]. Potent ABTS (2,2′-azino-bis-(3-ethylbenzothiazoline-6-sulfonic acid) diammonium salt) scavenging activity [14] as well as porcine pancreatic lipase inhibitory effects [15] were also reported. Starfruit is also rich in dietary fibers, especially insoluble ones [16]. Fiber-rich diets are reported to decrease the incidence of several diseases such as colorectal cancer [17].

With regard to its chemical composition, starfruit is known for its richness in phenolics [8,18] including flavonoid *C*-glycosides like carambolaflavone and carambolaside M [15]. Additionally, alkaloids of the tetrahydroisoquinoline group were isolated from starfruit such as (1R*,3S*)-1-(5-hydroxymethylfuran-2-yl)-3-carboxy-6-hydroxy-8-methoxyl-1,2,3,4-tetrahydroisoquinoline [19]. The volatiles of starfruit were extensively studied with 200 aroma components being previously reported [5]. Esters, acids, carbonyl compounds as well as aliphatic hydrocarbons and acids were the major previously identified classes [20,21,22]. In spite of all these health benefits, starfruit is considered as contraindicated in uremic patients due to its high oxalate content, regarded as an anti-nutritive [19]. It reduces the bioavailability of calcium and magnesium, disturbing the metabolism of the body’s absorption of these elements from the diet [23]. In addition, existence of excess oxalic acid in the diet enhances oxalate urolithiasis, osteoporosis, and arthritis [23]. Furthermore, starfruit was reported as being nephrotoxic, neurotoxic and to possess negative inotropic and chronotropic effects [13]. 

Considering that primary metabolites in starfruit mediate for its nutrient as well as its hazards, i.e.; oxalate, an objective of profiling its primary metabolome in an untargeted manner seems warranted to provide better insight into its metabolites composition. 

For food analysis, several modern approaches are increasingly adopted including that of large scale metabolomics aiming at the detailed characterization of metabolites within plant specimens [1]. Metabolomics mostly make use of hyphenated chromatographic techniques such as gas chromatography coupled generally with mass-spectrometry (GC/MS) [24], and data usually are further subjected to multivariate analyses [25] for visualization and samples classification. A mild and effective technique for volatiles characterization using GC/MS is its coupling to headspace solid phase micro-extraction (SPME). SPME excels over former methods, being solvent free and involving minor heat effect compared to steam distillation. Moreover, SPME enables the enrichment of volatiles from gas over a fused-silica (or other) fiber, followed by subsequent desorption of these analytes that leads to detection of less abundant volatiles [26]. 

Such a technique seems more suitable for starfruit aroma characterization due to its low volatiles content. Distortions of quantitative composition can occur in SPME, however, based on preferential absorption, or very rarely by reactions triggered by some absorbant materials like activated charcoal (not used herein). The addition of internal standard in sample matrix prior to aroma collection and measuring its recovery can help assess for technical variances in SPME analysis.

The objective of the current study was applying metabolomics tools for the first time to assess the metabolism of *A. carambola* in the context of both its geographical origin and ripening stages as well. *A. carambola* fruits derived from different origins viz., Indonesia and Malaysia as its endemic origin are compared to those cultivated in Egypt, and at different ripening stages for the latter specimen.

Due to the complexity of acquired data, multivariate data analysis was applied to ensure analytical rigorousness and classify fruit specimens. Furthermore, although volatile components of starfruit have been reported previously [5,20,22], this study can be considered the first one to assess its volatile profiles from different origins using the SPME technique as a cold volatiles collection method 

## 2. Results and Discussion

This study presents detailed metabolite profiles characterization of starfruit. Two analytical techniques were employed; GC-MS post silylation and SPME GC-MS targeting its primary metabolites and volatiles, respectively. 

### 2.1. Primary Metabolites Profiling Viz. Sugars, Amino and Organic Acids via GC-MS Post Silylation

**Figure 1:**
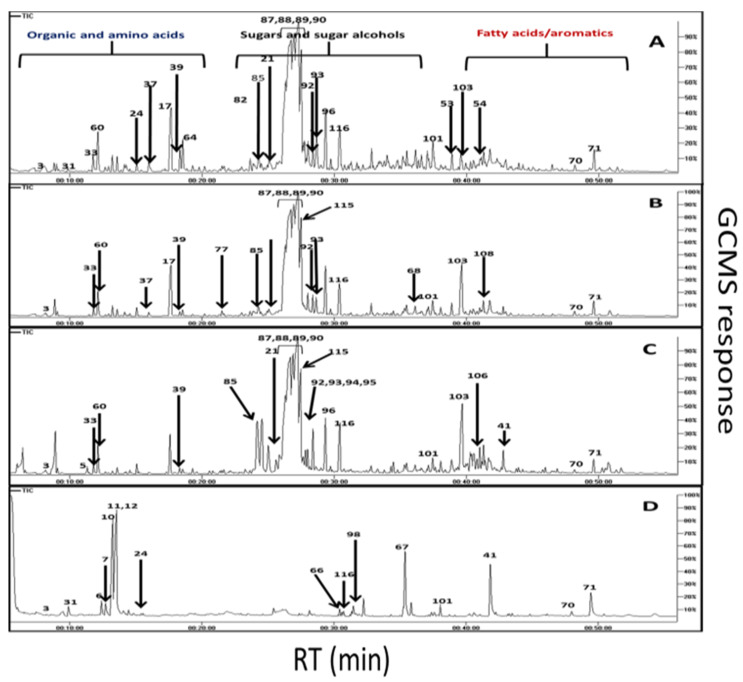
Gas chromatography coupled with mass-spectrometry (GC-MS) total ion chromatograms (TIC) of silylated metabolites in ripe fruit grown in Indonesia (**A**), Malaysia (**B**), Egypt (**C**) and unripe from Egypt (**D**).

To provide a comprehensive profile of *A. carambola* primary metabolites in the context of their geographical origin and ripening stage, GC-MS post silylation was employed (Figure 1). Two ripe fruits were collected from its endemic origins in Indonesia and Malaysia and were compared to those grown in Egypt. For Egyptian fruits, two stages were compared viz. unripe and ripe. A total of 117 volatile or volatilized metabolites were identified (Table 1) including low molecular weight sugars, polyols as well as acids, including amino and fatty acids, sterols, nitrogenous compounds and aromatics (phenolics, etc.).

#### 2.1.1. Sugars

Sugars were the most abundant metabolite class in all fruit specimens with ca. 33–86% relative abundance and were at much higher levels in ripe versus unripe fruits stage in the case of Egyptian fruits. Sugars serve as providers of carbon and as energy sources to humans in addition to their signaling role in plant homeostasis i.e., innate immunity [27]. Among detectable sugars, monosaccharides were the most abundant ones, represented by fructose (38–48%) followed by glucose (21–25%) in ripe fruits. Although high fructose levels are suggested to fuel the epidemic of type 2 diabetes [28], *A. carambola* L. flesh has been recommended for diabetics [29]. Such an effect is justified by the fruit’s low absolute content in fructose (compared to maize syrup), its richness in non-digestible sugar alcohols, and dietary fiber, especially insoluble fiber that has been shown to exhibit potential hypoglycemic effects [16]. All this poses *A. carambola* fruit as a low-calorie food with only 34 kcal/100 g [30]. 

#### 2.1.2. Polyols

The enrichment of sugar polyols in fruit specimens (3.5–11%) also justifies its low-calorie content exemplified by the abundance of acyclic polyols, i.e., sorbitol (2.4–10.5%) which is not absorbed from the intestine and does not lead to an increase in blood sugar levels. Sorbitol is used as a sugar substitute for diabetic patients and is commonly used in food manufacturing as a low cariogenic bulk sweetener in addition to its inclusion in foods and soft drinks [31].

Nevertheless, starfruit juice was reported to increase dentine permeability and to possess a strong erosive ability to remove the smear layer, suggesting its cautious consumption, especially in patients with dentine hypersensitivity [32]. Sorbitol showed the lowest levels in unripe Egyptian fruit (0.6%) versus the highest in Malaysian ripe fruit (10.3%). *Myo*-inositol, a common cyclic polyol was particularly enriched in the unripe fruit (5.6%) and at much lower comparable levels among ripened fruits (0.8–1.02%). *Myo*-inositol possesses an insulin-mimetic effects claimed to be an effective and safe strategy for improving glycemic control in type 2 diabetes [33]. Such results regarding sugar polyols fall in line with the reported use of starfruit as a hypoglycemic agent [13]. 

#### 2.1.3. Organic Acids

*A. carambola* L. are often categorized into sweet and sour classes [34], mostly attributed to differences in its organic acid content. Organic acids were found predominantly in the unripe Egyptian specimen (19.5%), whereas in the ripened fruit they decreased (2.5%, 4.2% and 5.2%), and are likely to account for the unripe fruit sour taste. Organic acids i.e., citric, tartaric, malic, and oxalic are reported to contribute to fruit acidity [32] Oxalic acid was the chief organic acid in all examined fruits (1.7–2.7%) and accounted for almost 30% to 60% of the organic acid pool, except for Malaysian fruit in which it was found at trace levels (0.07%). This might be attributed to the agricultural practices. For instance, *Triticum aestivum* L. produced oxalic acid upon the addition of organic residue while genetic engineering of oxalate decarboxylase in tomato modulated the acid pool, especially oxalate [35,36].

These results are in agreement with previous reports on the correlation between oxalic acid levels in starfruit and its stage of maturity [37], and warrant the monitoring of its levels for fruit safety assessment. 

#### 2.1.4. Amino Acids

Free amino acids were marginally abundant in all fruit specimens except for the unripe Egyptian fruit which exhibited the highest levels (7.4%). The decrease in amino acids has been reported in a detailed study in peach fruit revealing an overall decrease in the levels of the amino acids during ripening, to be used either as energy fuels or precursors for flavonoid biosynthesis, as is likely to occur in case of A. carambola fruit [38].

Identified amino acids included pyroglutamic acid (the cyclic lactam of glutamic acid) [39], non-essential amino acids viz. serine and alanine versus proline, valine, and threonine as essential amino acids. Pyroglutamic acid was the most abundant amino acid derivative in all fruit specimens and has been reported to improve memory due to aging or alcoholism besides its antidiabetic effect in type 2 diabetes [39].

#### 2.1.5. Alcohols

Alcohols only contributed to the primary metabolites pool of the unripe Egyptian fruit (5.9%), being found at trace levels only in all other fruit specimens (0.06–0.1%), suggesting them as markers for ripening stages in *A. carambola* fruits. Detected alcohols included ethylene glycol at ca. 0.02–1.17% and nona-ethylene glycol at ca. 0.02–2.24%.

#### 2.1.6. Fatty Acids/Hydrocarbons and Sterols

Considering that no acid hydrolysis step was performed, our fatty acid results represent only the free form and not the esterified ones. Fatty acids, hydrocarbons, and sterols were detected at low levels in all ripe fruits specimens, while unripe fruits showed relatively higher fatty acid and sterol content. Hydrocarbons were detected in all fruits (0.02–3.2%) with the highest level found in unripe Egyptian fruit. Whereas sterols were detected in all fruits (0.2–2.3%) with highest level detected in unripe Egyptian fruits. Saturated fatty acids i.e., myristic, palmitic and stearic acids amounted to the majority of fatty acid species in all samples, followed by unsaturated and omega-3 fatty acids i.e., octadecenoic, oleic and α-linolenic acids. Omega-6 acids, i.e., linoleic acid, showed the least contribution to fatty acids contents in all fruit specimens. Both omega-6 and omega-3 are metabolically and functionally distinct essential fatty acids, needed in the human diet to maintain a balance in optimal growth and development [40,41]. A low omega-6/omega-3 fatty acid ratio is considered favorable to reduce the risk of heart-related diseases [42]. Fatty acid levels in tested fruit specimens revealed low omega-6/omega-3 ratios ranging from 1:2.5 to 1:6, with the lowest ratio existing in tropically grown fruits, unripe Egyptian fruit, and a relatively higher ratio in the ripe Egyptian specimen. The correlation between ripening of fruits and simultaneous decrease in omega-6/omega-3 ratio has been reported in a detailed study in strawberry fruit likely to be the case in tropically grown A. carambola fruits [43]. These findings emphasize the nutritional value of starfruit as hypolipidemic food.

#### 2.1.7. Nitrogenous Compounds

Nitrogenous compounds were found predominantly in the unripe Egyptian specimen (8.2%), whereas in the ripened fruit it was reduced to reach 0.8–1.3%, represented mainly by Gamma-aminobutyric acid (GABA) and *N*-Acetyl glucosamine.

Gamma-aminobutyric acid (GABA) was detected in all fruits (0.3–1.6%) with the highest levels found in ripe Indonesian and unripe Egyptian fruits. GABA is known to exert a hypotensive effect that might mediate for the known hypotensive effect of starfruit [44]. Another major nitrogenous compound detected in unripe Egyptian fruit was *N*-acetyl-glucosamine (2.1%), though it was found at trace levels in ripe fruits classified as reducing sugar and to serve as building blocks of connective tissue [45].

#### 2.1.8. Aromatics

Aromatics were detected in all fruits (0.2–3.3%) with the highest levels found in unripe and ripe Egyptian fruits. Identified aromatics included catechin, thymol β-glucopyranoside, benzoic acid, and α-tocopherol. Catechin was the most abundant in all fruits (0.04–1.4%), with the highest level found in Egyptian ripe fruit and reported to possess potent radical scavenging and antioxidant activities [46]. The twofold increase in flavonoid content of catechin in ripe fruit versus unripe fruit confirms our hypothesis that amino acids are spurred towards the formation in *A. carambola* fruit upon ripening. The increase in flavonoids upon ripening has been previously reported in tomato fruits as well [47]. Thymol β-glucopyranoside also contributed to the aromatics pool of all fruits at ca. 0.02–0.8%. Benzoic acid was the chief aromatic acid in Egyptian unripe fruit at ca. 1.03%, however, found at trace levels in all other fruit specimens (0.01–0.04%). Benzoic acid is known to be involved in fruits ripening and this can account for the decrease in its level between ripe and unripe fruits [48]. The results suggest the reported antioxidant activity likely to be mediated by other phytonutrients, e.g., polyphenolics, are not detected using GC-MS [15], and warrant the employment of other tools i.e., LC-MS for future profiling. Variation in bioactives has been reported in *A. carambola* fruit at different ripening stages, affecting its antioxidant effect [49].

### 2.2. Mutlivariate Data Analyses of Primary Fruit Metabolites by GC-MS

**Figure 2:**
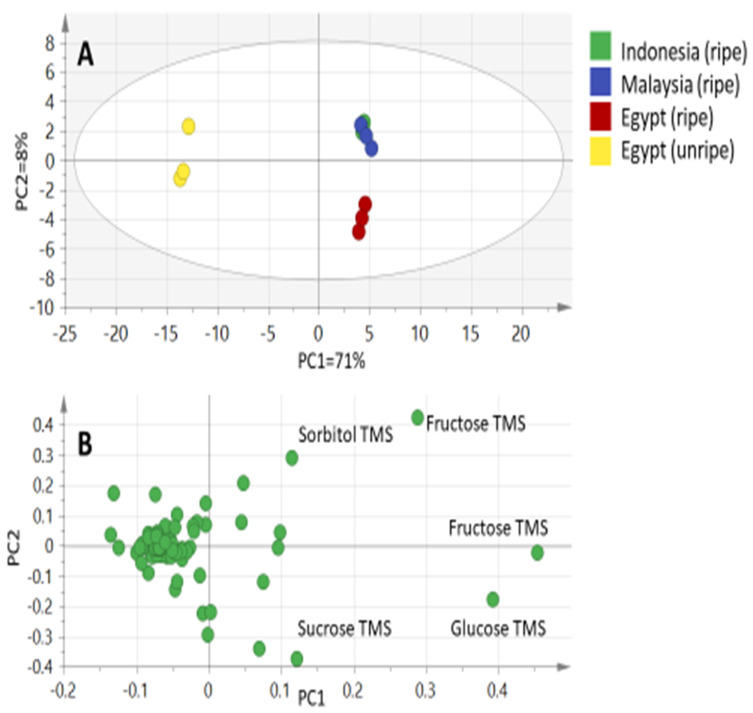
Principal component analyses of ripe and unripe silylated fruit extracts as analyzed by GC-MS (*n* = 3). (**A**) Score plot of PC1 vs. PC2 scores. (**B**) Loading plot for PC1 and PC2 contributing mass peaks and their assignments.

Although differences in chromatographic patterns were observed among fruit specimens, we attempted to categorize them in a holistic manner using chemometric tools, especially considering the relatively large number of 117 peaks in 4 fruit specimens, each represented by 3 replicates and totaling 12 samples. Principal component multivariate data analysis (PCA) was applied for the silylated fruit metabolites GC-MS abundance dataset (Figure 2A). It can explain 79% of the total variance described by PC1 and PC2. The PCA score plot (Figure 2A) reveals two clusters, with the unripe fruit specimens positioned with negative score values (left in PC1), whereas all other ripe fruit specimens clustered together with positive score values. Segregation between ripe Egyptian fruit from other ripe specimens also could be observed along PC2. Examination of the loading plot (Figure 2B) reveals that sorbitol, fructose, sucrose, and glucose contribute the most for specimen segregation, and are more abundant in all ripe fruit specimens. Sorbitol and fructose were more abundant in Malaysian and Indonesian specimens, versus sucrose and glucose found enriched in ripened Egyptian fruit. 

**Figure 3:**
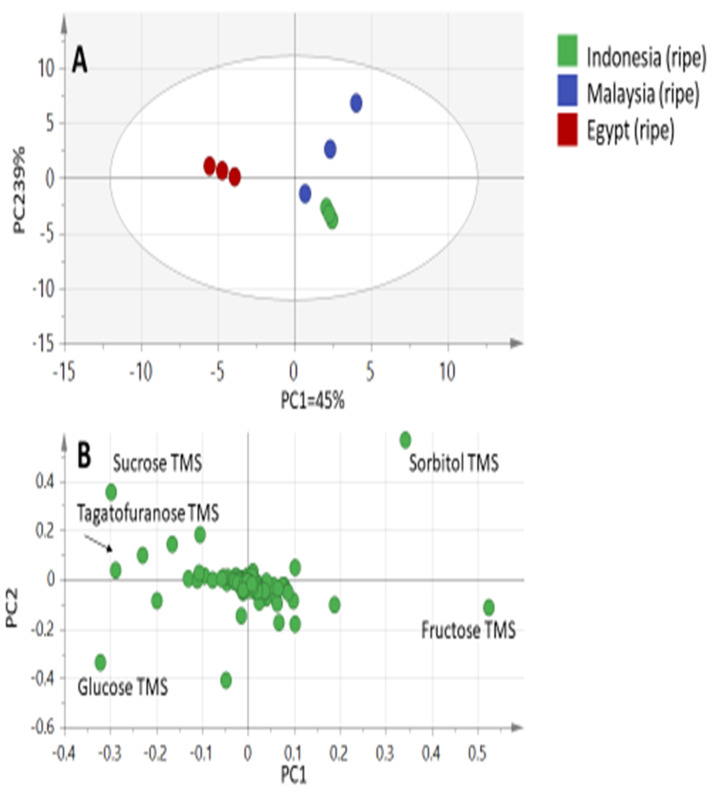
Principal component analyses of silylated ripe fruit extracts as analyzed by GC-MS (*n* = 3). (**A**) Score plot of PC1 vs. PC2 scores. (**B**) Loading plot for PC1 and PC2 contributing mass peaks and their assignments.

To help identify variation in the context of geographical origin, only ripe fruits from the three origins were modeled together in a second PCA trial (Figure 3A) excluding the unripe Egyptian ones. The main principal component (PC) to differentiate specimens in PCA, that is PC1, accounted for (only) 45% of the variance with ripe Egyptian fruit specimen being clustered at one side (negative PC1 score value) separable from Malaysian and Indonesian specimens positioned together on the other side (positive PC1). Examination of the loading plot (Figure 3B) reveals that variables corresponding to sugars viz. sucrose, tagatofuranose, and glucose, were found to be most enriched in Egyptian ripe fruit specimens. In contrast to this, sorbitol and fructose were more abundant in Malaysian and Indonesian fruits and are suggestive of the low-calorie claim, with the two tropically grown fruits being richer in polyols.

### 2.3. Volatiles Profiling via Headspace SPME Coupled to GC-MS 

Although starfruit aroma profiles were previously reported [20,22], this study is the first one to investigate volatile profiles within *A. carambola* fruits derived from different origins, especially comparing its endemic area grown fruits to those produced in Egypt. The unripe Egyptian fruit showed a very weak aroma profile as is typical in most fruits in which the flavor develops upon ripening and this is why it was not included in this part. Moreover, the volatiles cold extraction method used (SPME GC-MS), on one hand is less prone to distortion and artifact formation of analytes than others but may lead to selective enrichments on the other hand. Fruit volatiles—together with sugars/polyols—contribute most significantly to fruit aromas and flavor perception upon tasting and thus warrant careful characterization [50]. 

**Figure 4:**
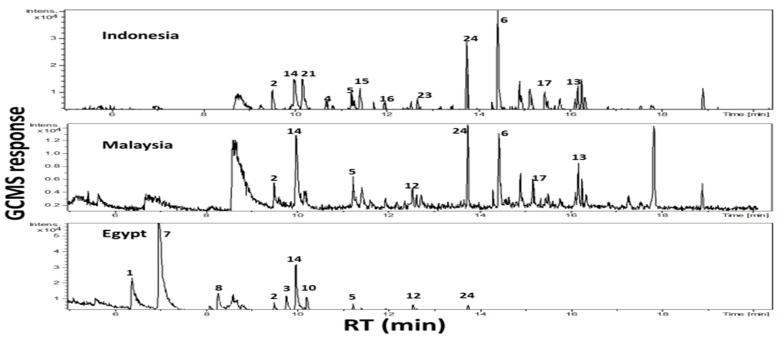
Headspace GC-MS total ion chromatograms (TIC) of volatile metabolites in ripe fruits grown in Indonesia, Malaysia and Egypt with peaks numbered followed that listed in Table 2.

Headspace GC-MS analysis led to the identification of 24 volatile constituents (Table 2), with fruits grown in Egypt showing the most distinct volatile profile compared to samples from endemic areas, and in agreement with primary metabolites analyses (Table 1). In contrast, Indonesian ripe fruit showed the largest number of peaks (Figure 4). Identified volatiles included esters, ketones, terpene hydrocarbons, acids as well as aldehydes.

#### 2.3.1. Esters

Volatile esters are flavor components in the majority of fruits. They serve as both cues for animal attraction and as protectants against pathogens in ripe fruits [51]. Esters amounted to the most abundant aroma class in Egyptian fruit (65.8%), found at much lower levels in Indonesian and Malaysian specimens (5.8–9.4%) suggestive of an impact of agricultural practice or growing conditions (soil, radiation, water) on fruit aromas. Lipids and amino acids are among the most likely precursors for ester production during fruit ripening and are considered to play an important role in determining both the levels and types of esters [51]. 

Such correlations fall in line with the presence of the highest amino acid levels in the unripe Egyptian fruit (Table 1). Methyl caproate (49.8%) and ethyl caproate (10.3%) have their highest levels in Egyptian fruit and are likely to contribute to its aroma. Oxalic acid esters are major volatiles in the Malaysian specimen (9.4%), followed by Indonesian (5.1%) and Egyptian (1.2%). Such results show that oxalic acid also is transformed into volatile derivatives and is not confined to primary metabolites (Table 1). This has implications if food safety is assessed by free oxalic acid analysis only.

#### 2.3.2. Aldehydes

Aldehydes mediate for the antibacterial and antioxidant activities of many essential oils [52] and many volatile ones are important flavor components, e.g., from lipid oxidation and breakdown. Major amounts are found in the Indonesian (41.6%) and Malaysian (38%) fruit specimens. Straight-chain aliphatic aldehydes were the most abundant ones, being present at 33.3% and 19.9% in Malaysian and Indonesian fruits, respectively. They likely serve as important food odorants in tropical grown *A. carambola* fruit [53]. Nonanal, the major straight-chain aliphatic aldehyde [54], was detected in all fruits at 13.8–33.5%. β-Cyclocitral, a woody or blue-green off-flavor compound [55] was the only aliphatic cyclic aldehyde detected almost exclusively in Indonesian fruit (4.9%). β- Cyclocitral is a norisoprenoid derived from carotenoids oxidation in *A. carambola* fruit, later accounts for fruit yellow color [56] and likely to serve as precursor of this aroma compound.

#### 2.3.3. Ethers/oxides

Myristicin is an aromatic ether present in many herbs used for flavorings and in spices [57]. Here it was found exclusively in tropically grown fruits and absent in Egyptian grown fruit. Safrole, another aromatic aldehyde was only detected in the Indonesian specimen (8.5%), and was absent from Malaysian and Egyptian fruits. Safrole is an important food-borne toxin with some evidence for genotoxic activity, with the US-FDA banning its use as a food additive [58]. Qualitative and quantitative differences in aroma composition among the same food of different origins is common and has been extensively reported from previous reports as these chemicals are produced in part adapting to plants own environment [59]. Variation in bioactives has also been reported in *A. carambola* fruit at different ripening stages and affecting its antioxidant effect [49].

#### 2.3.4. Ketones

Ketones constitute a major volatile class in Indonesian (23.6%) and Malaysian (25.4%) fruit specimens and to a lesser extent in the Egyptian ones (10.5%), exemplified by neryl acetone as a major component. 2-Hydroxy-2-methylhept-6-en-3-one existed in all three fruit specimens, though at varying levels (1.6–6.2%); 2-Nonanone was detected only in Egyptian fruit (2%). The presence of 2-nonanone in Egyptian fruits is likely to account for a sweet green weedy earthy flavor of its fruit but has yet to be confirmed using sensory analysis [60].

### 2.4. Quantitative Determination of Major Silylated Primary Metabolites 

The abundance of sugars (viz. glucose and fructose) as major metabolites in all fruit specimens, as well as the existence of the anti-nutrient oxalic acid warranted quantitative assessment of these major metabolites.

#### 2.4.1. Quantitative Determination of Major Detected Sugars Viz. Fructose and Glucose 

**Figure 5:**
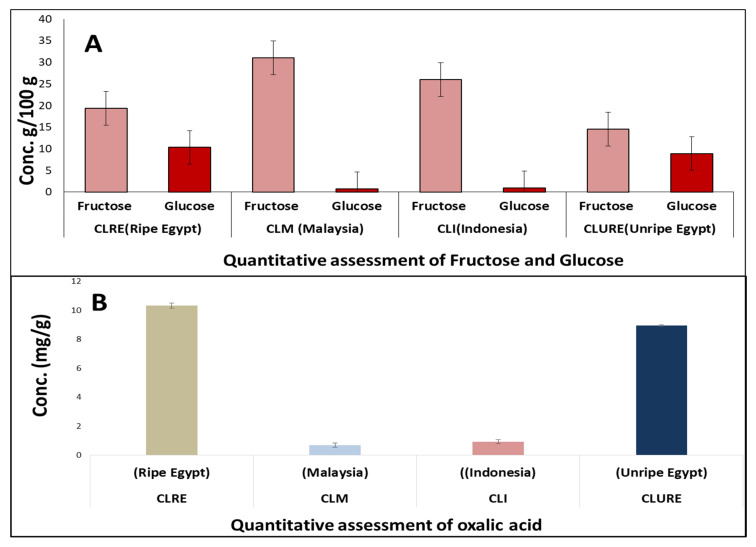
Quantitative assessment of major sugars and oxalic acid (*n* = 3). (**A**) Quantitative assessment of glucose and fructose (Conc. g/100g); (**B**) quantitative assessment of oxalic acid (Conc. mg/g).

A HPLC–RI method was adopted to determine glucose and fructose levels in fruit specimens from different origins (Figure 5A). 

**Figure 6:**
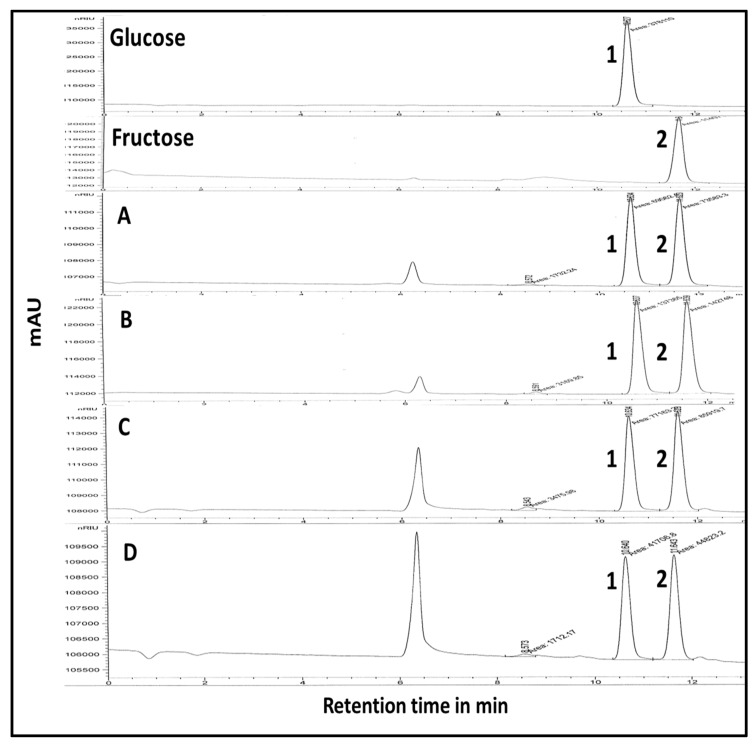
HPLC–RI chromatograms of the sugars of standard (glucose, RT = 10.62 min; fructose, RT = 11.64 min) and ripe fruit grown in Indonesia (**A**), Malaysia (**B**), Egypt (**C**) and unripe from Egypt (**D**).

Results revealed the highest fructose level in Malaysian fruit at (31.03g/100g fruit) followed by CLI (26.02), CLRE (19.4) and CLURE (14.6). These results are in line with the previously detected high prevalence of fructose in ripe fruits versus unripe (Figure 6). As for glucose level, CLRE showed highest level at (10.33 g/100g fruit) followed by CLURE (8.93), CLI (0.93) and CLM (0.70).

#### 2.4.2. Quantitative Determination of Oxalic Acid 

Likewise, absolute quantification of free oxalic acid revealed that the Egyptian fruits possessed the highest content at (10.3, 8.9 mg/g) for the ripe and unripe fruits, respectively. Much lower levels were observed in the case of the Indonesian and Malaysian fruits (0.9 and 0.7 mg/g, respectively) (Figure 5B).

### 2.5. Enzyme Inhibition Assays

To provide a biological comparative assessment of *A. carambola* fruits in the context of its geographical origin, alpha-glucosidase and pancreatic lipase inhibitory assays were performed. *A. carambola* L. flesh has been reported for the treatment of diabetes [29]. Moreover, pharmacological assays confirmed its hypoglycemic effect [13] as well as a porcine pancreatic lipase inhibitory effect [15]. Consequently, lipase and α-glucosidase were selected especially, being common targets in the pharmacotherapy of obesity and type II diabetes, with several inhibitors approved as anti-obesity and antidiabetic drugs [61]. α-Glucosidase inhibitors are one of the six classes of oral antidiabetic drugs used either alone or together with insulin, to treat diabetes [62].

#### 2.5.1. α-Glucosidase Inhibitory Assay

**Figure 7:**
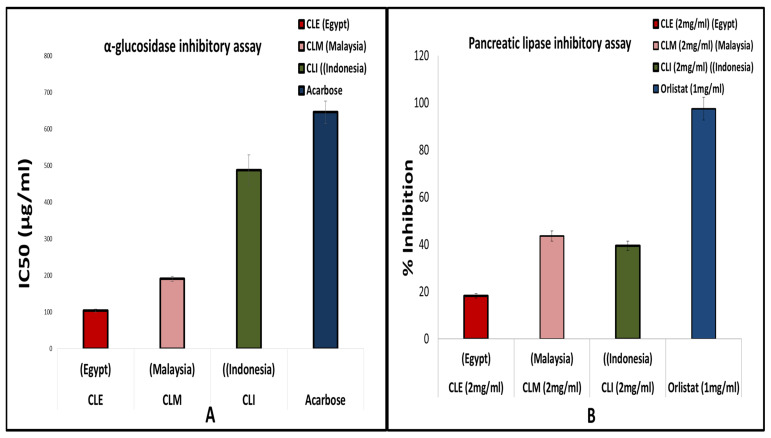
α-Glucosidase and pancreatic lipase inhibitory assays of ripe fruit extracts grown in Egypt (CLE), Malaysia (CLM), and Indonesia (CLI). (**A**) α-Glucosidase inhibitory assay, IC_50_ in (μg/mL) of fruit samples versus acarbose as positive drug control. (**B**) Pancreatic lipase inhibitory assay of fruit samples tested at 2 mg/mL compared to orlistat (at 1 mg/mL) (*n* = 3).

α-Glucosidase is a primary enzyme involved in carbohydrate digestion, regulating postprandial blood glucose levels in the human body. Its inhibitors are usually used to prevent or treat type II diabetes [63]. The three fruit samples of different origins i.e., CLE, CLM and CLI, were assessed for their α-glucosidase inhibitory activity. All tested fruit extracts exhibited strong inhibition, exceeding that of acarbose, a commercial α-glucosidase inhibitor anti-diabetic drug. IC_50_ values of the Egyptian, Malaysian and Indonesian fruits were determined at 104, 191 and 488 μg mL^−1^, respectively, compared to that of acarbose (646 μg mL^−1^) (Figure 7A). CLE exhibited the strongest effect whereas CLI was the least active fruit, as evident by its higher IC_50_ value. These findings are in line with samples richn in sugar polyols. However, sugar polyols were more enriched in CLM and CLI in discrepancy with the assay results, suggesting that α-glucosidase inhibition is most probably mediated by secondary metabolites rather than sugar polyols. 

#### 2.5.2. Pancreatic Lipase Inhibitory Assay 

*A. carambola* juice is reported to possess hypolipidemic activity [34]. The three fruit samples CLE, CLM and CLI were consequently assessed for their pancreatic lipase inhibitory activity at concentration 2 mg/mL. All tested fruit samples showed lower efficacy in lipase inhibition in comparison to orlistat (conc. 1 mg/mL) a potent competitive inhibitor of gastric and pancreatic lipase [64] (Figure 7B). Interestingly, CLE showed the least inhibition (18%) contrary to the effect observed on alpha-glucosidase, and suggestive of different constituents to mediate for either effect. Comparable inhibition levels were observed in the case of CLM and CLI (44% and 40%, respectively).

## 3. Materials and Methods 

### 3.1. Plant, SPME, and Chemicals

Four *A. carambola* fruit specimens including ripe fruits were collected from trees grown in Bogor, Indonesia, and commercial Malaysian fruits. For Egyptian fruits, two fruit specimens viz. unripe and ripe were collected in May from Groppy Arboretum, Giza, Egypt. As for the site of collection of the Egyptian fruits, the soil is of clay type, humidity is high and may reach 90%. The Egyptian tree is irrigated every 15 days and grows in shade. Fruits were immediately lyophilized and stored at −20 °C until further analysis. SPME holder and fiber (of length 2 cm) coated with 50 μm/30 μm DVB–CAR–PDMS (Divinylbenzene/Carboxen/Polydimethylsiloxane) was supplied by Supelco (Oakville, ON, Canada). All other chemicals and volatile standards were provided from Sigma Aldrich (St. Louis, MO, USA). Voucher specimens of fruits are deposited in the Pharmacognosy department, Faculty of Pharmacy, Cairo University (Giza, Egypt). 

### 3.2. SPME Volatiles Isolation from Whole Fruit

Headspace volatiles analysis using SPME was adopted from [65,66,67] with minor modifications. Briefly, whole lyophilized dried fruits were ground and 1.0 g was placed inside a 20 mL clear glass vial. (*Z*)-3-Hexenylacetate was spiked into the vials to serve as an internal standard (IS), dissolved in water to a final concentration of 1 µg/vial. Vials were then tightly capped and stored for 30 min at 50 °C with the SPME fiber inserted into the headspace above the fruit sample. Details on method optimization and limit of detection is fully described in [24,68]. The relative standard deviation (RSD) of retention time was in the range of 0.05–0.15%. The RSD of peak intensity varied between 2.63% and 8.08%.

### 3.3. GC-MS Volatile Analysis

SPME fibers were desorbed manually at 210 °C for 1 min in the injection port of a Shimadzu Model GC-17A interfaced with a Shimadzu model QP-5000 mass spectrometer (Japan). The HP quadrupole mass spectrometer was operated in the electron ionization mode at 70 eV with a scanning range set at *m*/*z* 40–500 and a source temperature of 180 °C. Isolation and identification of volatile components were done according to [69]. Peaks were first de-convoluted using AMDIS software (http://www.amdis.net) and identified by its retention indices (RI) relative to n-alkanes (C6-C20), mass spectrum matching to NIST, WILEY library database (>90% match) and with authentic standards (whenever available).

### 3.4. GC-MS Analysis of Silylated Primary Metabolites 

Analysis of primary metabolites followed the exact protocol detailed in our previous work [24,70,71,72]. 100 μL of 50% aqueous extract (prepared by extracting 100 mg of fruit skin and pulp in 5 mL 50% MeOH) was evaporated under nitrogen till dryness. Three independent biological replicates representing each fruit specimen were extracted and analyzed under identical conditions. For derivatization, 150 μL of *N*-methyl-*N*-(trimethylsilyl)-trifluoroacetamide (MSTFA) was added and incubated at 60 °C for 45 min. The samples were analyzed using GC-MS. Silylated derivatives were separated on Rtx-5MS (30 m length, 0.25 mm inner diameter, and 0.25 μm film) column. Injections (1 μL) were made in a (1:15) split mode, conditions: injector 280 °C, column oven 80 °C for 2 min, rate 5 °C/min to 315 °C, kept at 315 °C for 12 min. He carrier gas at 1 mLmin^−1^. The HP quadrupole mass spectrometer was operated in the electron ionization mode at 70 eV. The scan range was set at 50–650 *m*/*z*.

### 3.5. Quantitative Determination of Major Silylated Primary Metabolites Detected by GC-MS Analysis 

#### 3.5.1. Quantitative Determination of Oxalic Acid 

Quantitation of oxalic acid followed the protocol adopted in [73] with slight modifications. Whole lyophilized dried fruits were ground and 1 g was transferred to a 50 mL volumetric flask. HCl (30 mL 1M) was added and kept in boiling water bath for about 30 min. An amount of 0.5 mL of 5% calcium chloride was thoroughly mixed to precipitate oxalate. The mixture was then centrifuged for 15 min at 800 rpm. The supernatant was decanted and precipitate was washed with 2 mL 0.35 M ammonium hydroxide (NH_4_OH) and dissolved in 0.5 M sulfuric acid (H_2_SO_4_) and titrated against standardized 0.1 M potassium permanganate (KMnO4).

#### 3.5.2. Quantitative Determination of Major Detected SugarsGlucose and Fructose 

Quantitation of major sugars followed the exact protocol detailed in [74]. The lyophilized dried fruits were grinded and extracted with 100% H_2_O in an ultrasonic bath for 5 min, then filtered through PVDF 0.45 lm syringe filters. An aliquot of 1.5 mL was placed in vials for the analysis on an Agilent 1220 HPLC system. Three independent biological replicates representing each fruit specimen were extracted and analyzed under identical conditions. Standard solution for each sugar was prepared by dissolving the solid in Millipore water at a concentration range of 1352–1354 μg/mL. Analyses were performed using an Agilent 1220 HPLC system equipped with an HPXP, 9 μm column (300 × 7.8 mm, BIO-RAD, Hercules, CA, USA), and a refractive index detector (HPLC-RI). Analysis conditions were as follows: injection volume, 20 μL; mobile phase, Milli-Q grade H2O; flow rate, 0.7 mL/min; column temperature, 80 °C. The method validation was adopted according to [75].

### 3.6. GC-MS Multivariate Data Analyses

MS peak abundance of primary silylated metabolites were extracted using MET-IDEA software with default parameter settings for GC-MS [76]. The aligned peak abundance data table was further exported to principal component analysis (PCA) using the SIMCA-P version 13.0 software package (Umetrics, Umeå, Sweden). All variables were mean-centered and scaled to Pareto variance. 

### 3.7. Enzyme Inhibition Assays

#### 3.7.1. α-Glucosidase Inhibitory Assay 

The α-glucosidase inhibitory assay was measured in a 96-well microplate reader based on [77] with slight modification. In brief, 50 μL of each fruit sample at different concentrations (3.750, 1.875, 0.938, 0.469, 0.234, 0.118, 0.059, 0.029 and 0.015 mg/mL) dissolved in DMSO diluted in 50 μL 100 mM-phosphate buffer (pH 6.8) in a 96-well microplate, was mixed with 50 μL yeast α- glucosidase (2 U/mL) for 10 min prior to addition of 50 μL substrate (5 mM, *p*-nitrophenyl-α-d-glucopyranoside prepared in same buffer). The release of *p*-nitrophenol was measured at 405 nm spectrophotometrically 20 min post-incubation with substrate. Individual blanks for test samples were prepared, and acarbose was used as a standard inhibitor. Results are expressed as IC_50_ using the following Equation (1):

Inhibition (%) = [(Abs_control_ − Abs_sample_)/Abs_control_] × 100 (1)

#### 3.7.2. Pancreatic Lipase Inhibitory Assay 

Lipase inhibition was measured based on an assay described in reference [61] using a 96-well microplate reader. Each well contained 40 μL of the tested sample (2 mg/mL) and 40 μL of lipase type II from porcine pancreas (2.5 mg/mL prepared in Tris-Buffer (100 mM Tris × HCl and 5mM CaCl_2_, pH 7.0)). After preincubation for 15 min, 20 μL of 10mM p-nitro phenylbutyrate (pNPB) solution was added to each well followed by another incubation of 15 min at 37 °C. Absorbance was read at 405 nm. Orlistat was used as a positive inhibitor (1 mg/mL). Results are expressed as percentage of inhibition as samples were not enough to calculate an IC_50_ value for a tested concentration of 8 mg/mL. The inhibitory activity was calculated using the Equation (1) stated in Section 3.7.1.

### 3.8. Statistical Analysis

Results are presented as mean ± standard error of experiments performed in triplicates. IC_50_ values were calculated using nonlinear regression.

## 4. Conclusions

A multiplex approach combining HS-SPME coupled to GC-MS and GC-MS post silylation was adopted for metabolites profiling amongst *A. carambola* fruit specimens of four sample types: three geographically different origins and for one of these two ripening stages. To the best of our knowledge, this can be considered the first comprehensive attempt to compare volatile and primary metabolites compositional differences amongst *A. carambola* fruits of different origins and ripening stages.

Within the identified 117 primary metabolites, sugars expectedly were the most abundant ones and equally expected was that their increased content can be seen as the reason for the better taste of ripe fruits. However, the richness in sugar polyols and insoluble dietary fibers associated with a low-caloric intake and antidiabetic activity distinguishes starfruit from many other tropical fruits. Future work is needed in animals and more ideally in humans to determine glycemic index post ingestion of carambola fruit compared to other tropical fruit to confirm its low calorie value. Among the examined samples, Malaysian fruits revealed by far the most beneficial profile, being lower in caloric sugar and enriched in sorbitol. The Malaysian fruits appeared to be closest to the nutritional optimum composition with the lowest organic acid levels (2.6%) and concurrently with the highest ones in polyols (11.2%). However, to generalize this requires a wider sampling than was possible for this paper. Additionally, it had very low levels of undesired oxalic acid (but caution: higher levels of oxalate esters). From these analyses one could assume that Malaysian starfruit is nutritionally the most valuable one in various aspects, and as true as this is for the samples studied, this cannot be generalized yet, as the variety of genotypes and chemical phenotypes in the country of origin may be large and thus other cultivars from other farms in Malaysia might show much more negative profiles. In order to make a general statement, a large sampling effort of varied cultivars and producers will still be required. Thus the chief value of this study is the possibility of identifying future cultivars using the same metabolic profiling techniques of beneficial effects like; lower sugar with retained sweetness (maintained by polyols), almost absent oxalic acid and safrole content, and a rich volatiles profile.

This can be used for breeding and cloning selection, improving the nutritional and antidiabetic functional food aspects. Oxalic acid derivatives, exemplified by butyl propyl and heptyl propyl esters, however, were also detected in the aroma/fragrance components and volatiles, and thus can misguide safety assessments based on free acid contents only. 

A total of 24 volatile metabolites were detected in Egyptian fruits with methyl caproate as the major volatile component. Its absence from South-East-Asian fruit specimens suggests it as a candidate to serve as a marker for Egyptian starfruit—if verified in a broader more general study. The scent of tropically grown fruits was dominated by aldehydes/ethers, especially nonanal and myristicin, which were not detected in Egyptian fruits. 

We could show that the herein applied analytical platform allows us to study the influence of genotype, ripening stages, growth conditions (soil, climate, etc.) and agricultural practice as determinants for metabolite heterogeneity. However, further work needs to be performed by assessing more fruit origins to be more conclusive.

An extended approach utilizing liquid chromatography coupled to mass spectrometry (LC-MS) can be applied to pinpoint differences in bioactive secondary metabolite profiles among fruit accessions and to rationalize more *A. carambola* fruit taste and health effects, positive or detrimental, in more depth.

## Figures and Tables

**Table 1:** Primary metabolites analysis using GC-MS from ripe and Egyptian unripe *A. carambola* fruits with results expressed as a relative percentile of the total peak areas ± std. deviation (*n* = 3). (CLI) Indonesian ripe fruit, (CLM) Malaysian ripe fruit, (CLRE) Egyptian ripe fruit and (CLURE) Egyptian unripe fruit, *n* = 3.

Peak #	RT (min)	KI	Name	Class	CLI	CLM	CLRE	CLURE
1	7.34	1079	Lactic acid 2TMS*	Acid	0.39 ± 0.22	0.41 ± 0.05	0.17 ± 0.02	0.97 ± 0.69
2	7.69	1096	Glycolic acid 2TMS		0.01 ± 0.00	-	-	0.30 ± 0.39
3	8.58	1133	Oxalic acid 2TMS		1.97 ± 2.00	0.07 ± 0.00	2.71 ± 2.24	1.67 ± 0.33
4	10.45	1210	Malonic acid 2TMS		0.06 ± 0.00	0.02 ± 0.00	0.03 ± 0.01	0.82 ± 0.86
5	11.29	1242	Methylmalonate 2TMS		0.02 ± 0.01	0.02 ± 0.00	0.29 ± 0.05	1.29 ± 0.87
6	12.91	1305	Maleic acid 2TMS		0.26 ± 0.07	0.20 ± 0.04	0.07 ± 0.01	1.37 ± 0.81
7	13.21	1316	Succinic acid 2TMS		0.01 ± 0.00	-	-	0.74 ± 0.14
8	13.56	1330	Glyceric acid3TMS		0.02 ± 0.01	0.01 ± 0.00	0.01 ± 0.00	0.74 ± 0.14
9	13.85	1342	2,3-Dihydroxybutanoic acid 3TMS		0.01 ± 0.00	0.01 ± 0.00	-	0.66 ± 0.11
10	14.12	1352	Fumaric acid 2TMS		0.15 ± 0.02	0.11 ± 0.00	0.04 ± 0.00	1.12 ± 0.64
11	14.39	1362	Nonanoic acid, TMS ester		0.02 ± 0.01	0.02 ± 0.01	0.02 ± 0.00	1.51 ± 0.61
12	14.52	1367	Citramalic acid 2TMS ester		0.09 ± 0.02	0.11 ± 0.00	0.13 ± 0.01	1.07 ± 0.86
13	15.19	1393	Pentonic acid, 5-deoxy-3 TMS, γ-lactone		-	-	-	0.33 ± 0.44
14	15.54	1407	Glutaric acid 2TMS		-	-	-	0.68 ± 0.10
15	15.78	1417	2-Methylglutaric acid, 2TMS- ester		-	-	-	0.78 ± 0.10
16	16.03	1427	Erythronic acid γ-lactone, 2 TMS-ether		0.01 ± 0.01	-	-	0.77 ± 0.13
17	17.62	1490	Malic acid 3TMS*		1.89 ± 0.02	−1.41 ± 0.05	0.56 ± 0.05	0.65 ± 0.09
18	18.00	1506	Adipic acid 2TMS		-	-	-	1.34 ± 0.67
19	20.16	1595	Tartaric acid 2TMS-ether, 2TMS ester		0.06 ± 0.03	0.04 ± 0.01	-	0.63 ± 0.11
20	24.61	1794	Azelaic acid 2TMS		0.04 ± 0.00	0.01 ± 0.00	0.04 ± 0.00	0.92 ± 0.11
21	25.03	1814	Citric acid 4TMS*		0.14 ± 0.04	0.11 ± 0.04	0.12 ± 0.03	0.54 ± 0.32
22	27.63	1940	Ascorbic acid 4TMS-ether		-	-	-	0.65 ± 0.09
				Total acids	5.16	2.55	4.21	19.54
23	11.42	1247	Diethylene glycol, 2TMS	Alcohol	-	-	-	0.57±0.44
24	15.03	1387	Ethylene glycol 2TMS*		0.03 ± 0.01	0.02 ± 0.00	0.03 ± 0.00	1.17 ± 0.11
25	45.42	3047	Nonaethylene glycol 2TMS		0.05 ± 0.05	0.02 ± 0.02	0.03 ± 0.02	2.24 ± 0.40
26	46.61	3136	Octacosyl TMS		-	-	-	0.69 ± 0.10
27	49.24	3305	Decaethylene glycol 2TMS		-	-	-	0.68 ± 0.11
28	49.75	3332	1-Triacontanol TMS		0.02 ± 0.00	-	-	0.56 ± 0.25
				Total alcohols	0.10	0.06	0.07	5.91
29	14.68	1373	3,4-[(2)Trimethylsiloxy]dihydro-2(3*H*)-furanone	Aldehyde/furan	-	-	0.01 ± 0.00	0.64 ± 0.32
				Total furan	0.00	0.00	0.01	0.64
30	7.82	1102	Alanine 2TMS	Amino acid	0.06 ± 0.02	0.02 ± 0.00	0.02 ± 0.01	0.70 ± 0.14
31	9.83	1185	Proline TMS*		0.27 ± 0.05	0.07 ± 0.01	0.07 ± 0.01	0.16 ± 0.10
32	10.64	1217	Valine 2TMS*		0.05 ± 0.00	0.03 ± 0.00	0.02 ± 0.00	0.69 ± 0.16
33	11.75	1260	Serine 2TMS-		0.57 ± 0.00	0.26 ± 0.02	0.34 ± 0.05	0.59 ± 0.38
34	12.65	1295	Threonine, *O*-TMS, TMS ester		0.10 ± 0.03	0.04 ± 0.01	0.06 ± 0.01	1.19 ± 0.96
35	14.30	1359	Serine 3TMS		0.08 ± 0.07	-	-	0.67 ± 0.11
36	14.94	1384	Threonine 3TMS		0.01 ± 0.01	-	0.01 ± 0.00	0.66 ± 0.10
37	15.94	1423	Aspartic acid 2 TMS ester		0.35 ± 0.05	0.10 ± 0.03	0.04 ± 0.01	0.95 ± 0.18
38	17.16	1472	Proline TMS*		0.12 ± 0.00	0.02 ± 0.00	0.01 ± 0.00	0.78 ± 0.11
39	18.29	1518	Pyroglutamic acid 2TMS		1.51 ± 0.18	0.35 ± 0.02	0.57 ± 0.20	1.01 ± 0.41
				Total amino acids	3.12	0.89	1.15	7.40
40	11.60	1254	Benzoic acid TMS	Aromatic	0.04 ± 0.03	0.01 ± 0.00	0.01 ± 0.00	1.03 ± 0.51
41	42.74	2849	Catechin, 5TMS		0.04 ± 0.04	0.74 ± 0.20	1.42 ± 0.02	0.69 ± 0.11
42	43.34	2892	Thymol-β-glucopyranoside-*O*-TMS		0.08 ± 0.02	0.02 ± 0.01	0.05 ± 0.01	0.79 ± 0.14
43	46.49	3127	α-Tocopherol, *O*-TMS		-	0.01 ± 0.01	-	0.77 ± 0.12
				Total aromatics	0.17	0.79	1.47	3.28
44	25.72	1847	Myristic acid TMS	Free fatty acid	0.11 ± 0.00	0.11 ± 0.01	0.14 ± 0.01	0.41 ± 0.35
45	29.70	2047	Palmitic acid TMS*		0.02 ± 0.01	0.02 ± 0.00	0.02 ± 0.00	0.81 ± 0.09
46	30.74	2102	Methyl 8-octadecenoate		-	-	-	0.90 ± 0.76
47	31.23	2129	Methyl stearate		-	-	-	0.51 ± 0.53
48	32.64	2206	Linoleic acid TMS*		0.01 ± 0.00	0.01 ± 0.00	0.02 ± 0.00	0.66 ± 0.11
49	32.75	2213	α-Linolenic acid TMS		0.03 ± 0.02	0.03 ± 0.00	0.03 ± 0.00	0.87 ± 0.12
50	32.76	2214	Octadecenoic acid TMS		0.02 ± 0.01	0.02 ± 0.01	0.01 ± 0.00	0.73 ± 0.10
51	32.88	2221	Oleic acid TMS		0.01 ± 0.01	0.01 ± 0.00	0.01 ± 0.00	1.17 ± 0.28
52	33.23	2241	Stearic acid TMS*		0.06 ± 0.02	0.05 ± 0.00	0.06 ± 0.00	0.88 ± 0.51
53	38.84	2583	1-Monopalmitin TMS		0.09 ± 0.05	0.08 ± 0.03	0.06 ± 0.00	0.47 ± 0.41
54	41.70	2775	Stearic acid, 2,3-[2trimethylsiloxy]propyl ester		0.06 ± 0.03	0.06 ± 0.01	0.05 ± 0.01	0.65 ± 0.82
				Total free fatty acids	0.42	0.40	0.39	8.06
55	34.29	2301	Tricosane	Hydrocarbon	0.01 ± 0.01	-	-	0.82 ± 0.02
56	37.56	2500	Pentacosane		-	-	-	0.33 ± 0.45
57	39.09	2599	Hexacosane		0.04 ± 0.01	0.02±0.00	0.02 ± 0.01	0.70 ± 0.10
58	40.60	2699	Heptacosane		0.02 ± 0.00	-	0.04 ± 0.00	0.68 ± 0.09
59	43.43	2898	Nonacosane		0.01 ± 0.00	-	0.01 ± 0.00	0.66 ± 0.10
				Total hydrocarbons	0.08	0.03	0.06	3.20
60	12.11	1274	Phosphoric acid 3TMS	Inorganic	2.31 ± 0.44	1.85 ± 0.46	2.05 ± 0.20	0.64 ± 0.10
				Total inorganic	2.31	1.85	2.05	0.64
61	9.14	1156	2-Pyrrolidinone TMS	Nitrogenous	0.02 ± 0.01	-	0.01 ± 0.00	0.64 ± 0.44
62	11.57	1253	Urea 2TMS		0.01 ± 0.01	-	0.02 ± 0.00	0.66 ± 0.11
63	11.85	1264	Aminoethanol, *O*,*N*,*N*-3 TMS		0.13 ± 0.00	0.04 ± 0.00	0.10 ± 0.02	0.58 ± 0.39
64	18.50	1526	GABA 3TMS		1.58 ± 0.51	0.36 ± 0.05	0.34 ± 0.07	0.80 ± 0.08
65	21.85	1669	unknown nitrogenous		0.01 ± 0.00	0.01 ± 0.00	0.01 ± 0.00	0.68 ± 0.09
66	30.18	2072	*N*-Acetyl-d-glucosamine 4 TMS		0.35 ± 0.44	0.65 ± 0.01	0.08 ± 0.03	2.09 ± 3.21
67	35.28	2360	Oleic acid amide		0.01 ± 0.00	0.01 ± 0.00	0.01 ± 0.00	1.02 ± 0.29
68	36.10	2409	Oleamide, *N*-TMS		0.33 ± 0.09	0.23 ± 0.02	0.25 ± 0.07	1.47 ± 0.23
69	37.41	2490	(*E*)-13-Docosenamide		0.01 ± 0.00	0.02 ± 0.00	0.02 ± 0.00	0.27 ± 0.42
				Total nitrogenous	2.45	1.32	0.84	8.22
70	48.12	3237	Campesterol TMS	Sterol	0.07 ± 0.00	0.06 ± 0.03	0.05 ± 0.01	1.18 ± 0.52
71	49.55	3322	β-Sitosterol TMS*		0.18 ± 0.02	0.14 ± 0.02	0.16 ± 0.01	1.13 ± 0.84
				Total sterols	0.25	0.19	0.21	2.31
72	16.67	1452	Threose 3TMS	Sugar	-	-	-	0.80 ± 0.13
73	18.83	1540	Threonic acid 3TMS		-	-	-	0.63 ± 0.11
74	19.25	1558	Erythronic acid 4TMS		0.06 ± 0.04	0.04 ± 0.01	0.11 ± 0.00	0.63 ± 0.10
75	20.54	1611	Ribofuranose 4TMS (isomer 1)		0.03 ± 0.00	0.03 ± 0.00	0.09 ± 0.01	0.76 ± 0.13
76	20.79	1622	Arabinonic acid, 3TMS γ-lactone		-	-	0.01 ± 0.00	0.63 ± 0.11
77	21.47	1652	Lyxose 4TMS		0.12 ± 0.00	0.14 ± 0.01	0.08 ± 0.01	0.76 ± 0.13
78	21.63	1659	Arabinose 4TMS		0.06 ± 0.01	0.03 ± 0.01	0.06 ± 0.00	0.63 ± 0.10
79	22.07	1679	Xylose 4TMS		0.03 ± 0.00	0.02 ± 0.00	0.06 ± 0.01	0.77 ± 0.12
80	22.25	1686	Mannose, 6-deoxy-2,3,4,5-4-*O*-TMS		0.02 ± 0.02	-	0.01 ± 0.00	0.64 ± 0.10
81	22.49	1697	Levoglucosan 3TMS		0.01 ± 0.00	0.01 ± 0.00	0.02 ± 0.00	0.79 ± 0.12
82	22.81	1711	Rhamnose 4TMS		0.11 ± 0.00	0.06 ± 0.01	0.07 ± 0.01	2.84 ± 0.93
83	22.97	1718	Fucose 4TMS		0.03 ± 0.00	0.02 ± 0.00	0.01 ± 0.00	0.64 ± 0.10
84	23.94	1763	Sorbofuranose 5TMS		-	-	0.10 ± 0.02	0.65 ± 0.11
85	24.22	1776	Tagatofuranose 5TMS		1.21 ± 0.13	1.18 ± 0.01	3.69 ± 0.40	0.59 ± 0.47
86	24.69	1797	Fructofuranose 5TMS		0.17 ± 0.15	0.38 ± 0.34	2.16 ± 1.98	0.86 ± 0.05
87	26.13	1866	Fructose 5TMS*		31.45 ± 1.01	28.68 ± 3.44	29.96 ± 1.35	0.65 ± 0.09
88	26.64	1891	Glucose 5 TMS*		23.08 ± 1.02	20.73 ± 4.26	24.93 ± 0.29	0.43 ± 0.36
89	26.77	1897	Fructose 5TMS*		16.47 ± 0.05	15.95 ± 0.00	8.48 ± 0.54	0.32 ± 0.35
90	26.98	1908	Sorbose 5TMS		1.14 ± 0.01	1.08 ± 0.08	0.85 ± 0.26	0.64 ± 0.10
91	26.99	1908	Glucose 5 TMS*		0.03 ± 0.01	0.02 ± 0.01	0.05 ± 0.01	0.67 ± 0.10
92	28.35	1977	Gulose, 5TMS		0.63 ± 0.12	0.61 ± 0.10	0.62 ± 0.04	0.87 ± 0.76
93	28.41	1980	Mannose 5TMS		0.96 ± 0.65	1.46 ± 0.83	2.05 ± 0.08	0.27 ± 0.24
94	28.57	1988	Galactose 5TMS		0.52 ± 0.14	0.24 ± 0.04	0.02 ± 0.00	0.66 ± 0.08
95	28.62	1990	Gluconic acid 6TMS		0.59 ± 0.08	0.31 ± 0.06	0.25 ± 0.04	0.54 ± 0.30
96	29.25	2023	Allose 5TMS-ether TMS		2.44 ± 0.11	2.36 ± 0.06	2.12 ± 0.30	0.87 ± 0.11
97	30.71	2100	Sedoheptulose 6TMS		0.04 ± 0.00	0.03 ± 0.00	0.13 ± 0.01	1.01 ± 0.50
98	31.13	2123	Sedoheptulose 6TMS		0.10 ± 0.01	0.03 ± 0.01	0.04 ± 0.00	1.58 ± 1.34
99	31.79	2159	Fructopyranose, 4TMS		0.05 ± 0.01	0.01 ± 0.00	-	0.73 ± 0.10
100	32.20	2182	Glucopyranose, 5TMS		0.11 ± 0.03	0.03 ± 0.01	0.05 ± 0.01	0.86 ± 0.12
101	37.50	2496	*O*-β-Galactopyranosyl-d-mannopyranose 8TMS		0.31 ± 0.04	0.21 ± 0.04	0.13 ± 0.02	0.62 ± 0.31
102	38.98	2591	Lactose 8TMS		0.01 ± 0.00	0.02 ± 0.01	0.01 ± 0.00	0.66 ± 0.10
103	39.50	2626	Sucrose 8TMS*		0.64 ± 0.18	5.79 ± 0.35	7.28 ± 0.39	0.77 ± 0.13
104	40.24	2675	Mannobiose 8TMS		0.30 ± 0.01	0.22 ± 0.01	0.76 ± 0.07	0.87 ± 0.24
105	40.59	2698	Cellobiose 8TMS		0.09 ± 0.05	0.05 ± 0.00	0.06 ± 0.00	0.86 ± 0.13
106	40.95	2723	Sucrose 8TMS*		0.35 ± 0.06	0.24 ± 0.02	0.65 ± 0.09	0.79 ± 0.14
107	41.05	2731	Trehalose 8TMS		0.15 ± 0.04	0.07 ± 0.01	0.05 ± 0.00	0.97 ± 0.22
108	41.25	2744	Maltose 8TMS		0.31 ± 0.06	0.38 ± 0.01	0.69 ± 0.07	0.56 ± 0.29
109	42.25	2814	Gentiobiose 8TMS		0.12 ± 0.01	0.08 ± 0.00	0.10 ± 0.01	0.86 ± 0.15
110	42.27	2816	Isomaltulose 7TMS		0.14 ± 0.01	0.06 ± 0.01	0.07 ± 0.00	0.80 ± 0.15
111	42.91	2861	Gentiobiose 8TMS		0.19 ± 0.01	0.10 ± 0.01	0.16 ± 0.01	0.82 ± 0.13
112	45.03	3018	unidentified disaccharide		0.01 ± 0.00	-	-	1.32 ± 0.20
				Total sugars	82.07	80.68	85.96	32.68
113	17.81	1498	Threitol 4TMS	Polyol	0.01 ± 0.00	-	-	0.47 ± 0.39
114	23.32	1735	Fucitol 5TMS		0.03 ± 0.00	0.02 ± 0.00	0.02 ± 0.00	0.54 ± 0.50
115	27.40	1929	Sorbitol 6TMS		2.88 ± 0.20	10.35 ± 7.10	2.43 ± 0.07	0.66 ± 0.08
116	30.39	2083	*Myo*-Inositol 6TMS		0.80 ± 0.21	0.82± 0.07	1.02 ± 0.08	5.57 ± 7.45
117	43.89	2932	Galactinol 9TMS		0.14 ± 0.00	0.03 ± 0.00	0.11 ± 0.01	0.90 ± 0.15
				Total polyols	3.87	11.23	3.58	8.13
			Total 100%		100.00	100.00	100.00	100.00

KI: Kovats retention index; * Denotes metabolites confirmed by matching with standard.

**Table 2:** Volatiles composition using solid phase micro-extraction (SPME) GC-MS of different origins (CLI) Indonesian ripe fruit, (CLM) Malaysian ripe fruit and (CLRE) Egyptian ripe fruit expressed as average relative percentile.

Peak #	RT (min)	RI	Name	Class	CLRE	CLI	CLM
1	6.41	873	2-Heptanone	Ketone	5.69	-	-
2	9.52	1051	2-Hydroxy-2-methylhept-6-en-3-one		1.61	6.17	3.69
3	9.78	1068	2-Nonanone		2.00	-	-
4	10.70	1129	4-Ketoisophorone		-	2.55	-
5	11.24	1167	3-Hexanone, 2,4-dimethyl-		1.25	2.73	9.02
6	14.43	1415	Nerylacetone		-	12.13	12.72
				Total ketones	10.55	23.58	25.44
7	7.00	902	Methyl caproate	Ester	49.82	-	-
8	8.29	976	Ethyl caproate		10.32	-	-
9	10.11	1089	3-Hexen-1-ol, propanoate, (*Z*)-		0.22	-	-
10	10.23	1097	Methyl caprylate		4.23	-	-
11	11.96	1219	Linalyl acetate		-	0.69	-
12	12.54	1263	Oxalic acid, butyl propyl ester		1.20	1.30	2.91
13	16.17	1548	Oxalic acid, heptyl propyl ester		-	3.84	6.49
				Total esters	65.79	5.83	9.40
14	9.99	1081	Nonanal	Aldehyde	16.49	13.82	33.46
15	11.43	1181	Decanal		0.10	6.16	-
16	11.73	1202	β-Cyclocitral		-	4.89	-
17	15.45	1499	Myristicin		-	16.82	4.61
				Total aldehydes	16.59	41.69	38.07
18	8.11	965	Myrcene	Monoterpene hydrocarbon	0.46	-	-
19	8.82	1006	Limonene		1.40	-	-
				Total monoterpene hydrocarbons	1.86	-	-
20	8.62	994	Hexanoic acid	Acid	3.85	-	-
				Total acids	3.85	-	-
21	10.18	1094	2,5-dimethyl cyclohexanol	Alcohol	-	9.41	-
				Total alcohols	-	9.41	-
22	12.63	1270	(*E*)-Anethole	Oxide	0.45	-	-
23	12.68	1274	Safrole		-	8.53	-
				Total oxides	0.45	8.53	-
24	13.75	1360	Tridecane, 4-methyl-	Aliphatic hydrocarbon	0.92	10.97	18.07
				Total aliphatic hydrocarbons	0.92	10.97	18.07
Total					99.56	100.00	90.98
